# Molecular determinants of post-mastectomy breast cancer recurrence

**DOI:** 10.1038/s41523-018-0089-z

**Published:** 2018-10-12

**Authors:** Kimberly S. Keene, Tari King, E. Shelley Hwang, Bo Peng, Kandace P. McGuire, Coya Tapia, Hong Zhang, Sejong Bae, Faina Nakhlis, Nancy Klauber-Demore, Ingrid Meszoely, Michael S. Sabel, Shawna C. Willey, Agda Karina Eterovic, Cliff Hudis, Antonio C. Wolff, Jennifer De Los Santos, Alastair Thompson, Gordon B. Mills, Funda Meric-Bernstam

**Affiliations:** 1 Department of Radiation Oncology, University of Alabama at Birmingham, Birmingham, AL 35249 UK; 2 Department of Surgery, Dana Farber Cancer Institute, Boston, MA 02215 USA; 3 Department of Surgery, Duke University, Durham, NC 27710 USA; 4 Department of Bioinformatics and Computational Biology, University of Texas MD Anderson Cancer Center, Houston, TX 77030 USA; 5 Department of Surgery, Massey Cancer Center, Virginia Commonwealth University, Richmond, VA 23298 USA; 6 Department of Translational Molecular Pathology, University of Texas MD Anderson Cancer Center, Houston, TX 77030 USA; 7 Department of Pathology, Memorial Sloan Kettering Cancer Center, New York, NY 10065 USA; 8 Department of Statistics, University of Alabama at Birmingham, Birmingham, AL 35249 UK; 9 Department of Surgery, Medical University of South Carolina, Charleston, SC 29425 USA; 10 Department of Surgery, Vanderbilt University, Nashville, TN 37204 USA; 11 Department of Surgery, University of Michigan Ann Arbor, Ann Arbor, MI 48109 USA; 12 Department of Surgery, Georgetown, Washington, DC 20007 USA; 13 Department of Pathology, University of Texas MD Anderson Cancer Center, Houston, TX 77030 USA; 14 Department of Oncology, Memorial Sloan Kettering Cancer Center, New York, NY 10065 USA; 15 Department of Hematology and Oncology, John Hopkins University, Baltimore, MD 21287 USA; 16 Department of Surgery, University of Texas MD Anderson Cancer Center, Houston, TX 77030 USA

## Abstract

Breast cancer (BC) adjuvant therapy after mastectomy in the setting of 1–3 positive lymph nodes has been controversial. This retrospective Translational Breast Cancer Research Consortium study evaluated molecular aberrations in primary cancers associated with locoregional recurrence (LRR) or distant metastasis (DM) compared to non-recurrent controls. We identified 115 HER2 negative, therapy naïve, T 1–3 and N 0-1 BC patients treated with mastectomy but no post-mastectomy radiotherapy. This included 32 LRR, 34 DM, and 49 controls. RNAseq was performed on primary tumors in 110 patients; with no difference in RNA profiles between patients with LRR, DM, or controls. DNA analysis on 57 primary tumors (17 LRR, 15 DM, and 25 controls) identified significantly more *NF1* mutations and mitogen-activated protein kinase (MAPK) pathway gene mutations in patients with LRR (24%, 47%) and DM (27%, 40%) compared to controls (0%, 0%; *p* < 0.0001 and *p* = 0.0070, respectively). Three patients had matched primary vs. LRR samples, one patient had a gain of a *NF1* mutation in the LRR. There was no significant difference between the groups for *PTEN* loss or cleaved caspase 3 expression. The mean percentage Ki 67 labeling index was higher in patients with LRR (29.2%) and DM (26%) vs. controls (14%, *p* = 0.0045). In summary, mutations in the MAPK pathway, specifically *NF1*, were associated with both LRR and DM, suggesting that alterations in MAPK signaling are associated with a more aggressive tumor phenotype. Validation of these associations in tissues from randomized trials may support targeted therapy to reduce breast cancer recurrence.

## Genomics: NF1 mutations more frequent in recurrent breast cancer

Women with breast cancer who relapse following surgery frequently harbor genomic alterations in a pathway linked to cell proliferation and anti-tumor immune responses. A USA-based team led by Funda Meric-Bernstam from the University of Texas MD Anderson Cancer Center in Houston, and Kimberly Keene from the University of Alabama at Birmingham conducted a thorough molecular analysis of tumor samples obtained from 115 women with 5 years of follow-up after a mastectomy and, oftentimes, adjuvant chemotherapy or hormonal therapy. RNA profiles did not significantly differ between women who experienced local recurrence, developed distant metastases or were still in remission. However, DNA analyses identified significantly more mutations in patients whom recurred in the mitogen-activated protein kinase cell signaling pathway, specifically NF1, implicated in cell growth and immunity. If validated, the findings support targeting this pathway to prevent disease recurrence.

## Introduction

Breast cancer represents a heterogeneous disease process with markedly different treatment outcomes despite similar clinical staging.^1–3^ Even with the most aggressive and modern therapy, as much as 15% of patients develop local-regional recurrences (LRR) which in turn increase the risk of distant disease and death.^4–6^ The population who remains at increased risk of a LRR may benefit from either more intensive therapy, such as an increased radiation boost dose, addition of a radiation sensitizer, an increase in the volume irradiated, such as the addition of a supraclavicular field, or additional targeted systemic therapy.

An area of specific controversy is the management of post-mastectomy patients with 1–3 positive nodes. The relative risk of LRR in this setting has been controversial and the benefit of radiation is felt to be minimal in many of these patients. Yet, radiation therapy is increasingly offered to post-mastectomy patients with 1–3 positive nodes, with potential radiation-related morbidity.^7^ In spite of the greatly variable practice in this area, a randomized trial testing the efficacy of post-mastectomy radiation failed to adequately accrue. The use of radiation in this setting also has important implications for surgical planning (e.g. utilization of immediate breast reconstruction) and is a significant economic burden on the health care system. Therefore, it is crucial that we find solutions to best utilize treatment modalities for our patients by identifying patients at greater risk for LRR.

Oncologists are increasingly using predictive factors and molecular subtype classifications to guide systemic treatment decisions.^8,9^ Individual risk factors such as age, tumor size, and grade are clinically limited in ability to predict outcome; therefore, the identification of a molecular profile to more accurately assess risk remains an important goal. A molecular profile that would sensitively and specifically address the risk of LRR would be important to determine which patients are at low risk of LRR in the absence of post-mastectomy radiation, in which the toxicity and inconvenience of radiation therapy may be avoided. Increased cell proliferation, and/or decrease in apoptosis are also indicators of more aggressive tumor biology. Therefore, Ki-67, as a measure of cell proliferation, cleaved caspase 3 (CC3), as an indicator of apoptosis, and PTEN, a tumor suppressor known to play an important role in breast cancer biology, were also evaluated specifically as a possible and simple means of predicting those at increased risk of recurrence.

This study was designed through the Translational Breast Cancer Research Consortium (TBCRC). The primary goal was to evaluate the molecular alterations associated with any recurrence, either LRR or DM, and compare them to a control group who did not have a recurrence. Furthermore, we sought to determine whether molecular predictors exist specific for locoregional recurrence compared to distant recurrence.

## Results

**Fig. 1:**
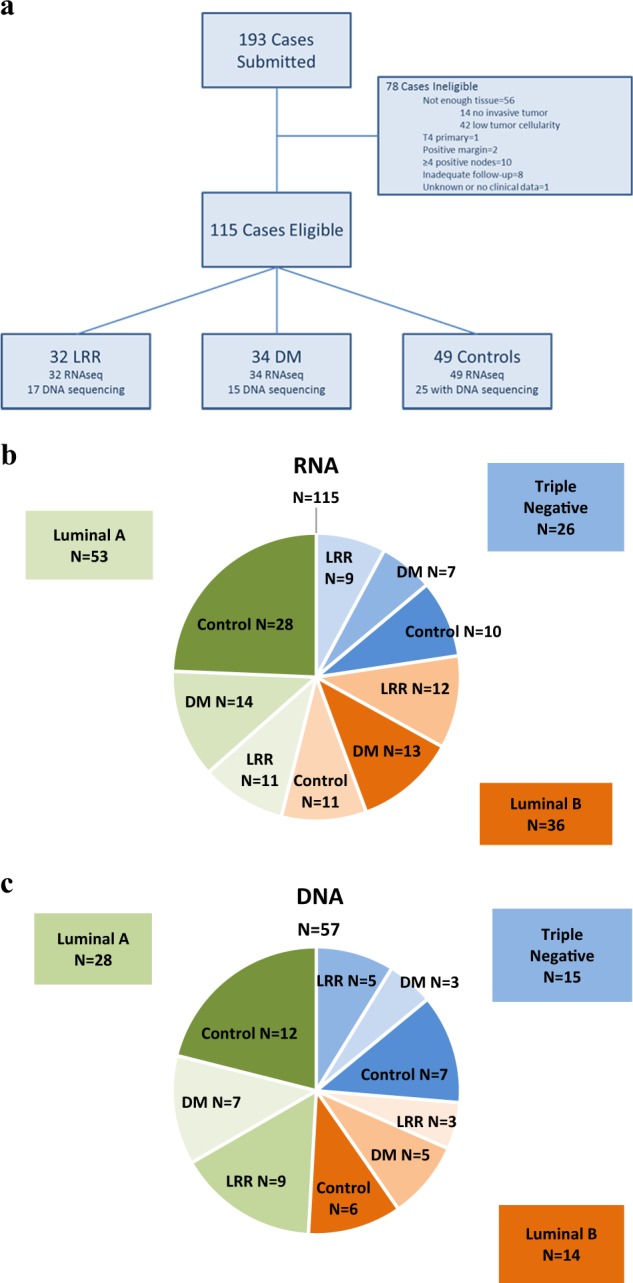
**a** Case collection and assay completion. **b** RNA mutational analysis case collection by tumor subtype. **c** DNA mutational analysis case collection by tumor subtype

A total of 193 FFPE samples were received from 10 TBCRC institutions. Of the 193, 78 were ineligible due to inadequate tumor cellularity (Fig. 1a–c). The remaining 115 patients included 32 LRR, 34 DM, and 49 controls. Age ranged from 25 to 88 years with a mean age of 52 years at diagnosis. Notably, although often one of the samples of each case-control pair submitted had inadequate cellularity, the demographic and clinicopathological characteristics were similar among the evaluable patients with LRR, DM, and control patients (Table 1). Neoadjuvant therapy was an exclusion criterion. Adjuvant therapy included chemotherapy in 81 patients, 63 of which were treated with anthracycline-based regimens. Adjuvant endocrine therapy was given in 72 out of the 87 patients who were ER positive and 48 of these patients were treated with tamoxifen. There were 95 white/Caucasian patients, 16 African Americans, and 1 Hispanic patient. Within the 115 patients there were 26 total hormone negative (triple negative) cases evenly distributed among the LRR, DM, and control cohorts. The median follow-up time for controls was 105 months (range 62–184 months). The median time to LRR was 34.6 months (range 6.6–127.6 months). The median time to DM was 35.2 months (range 4.4–96.2 months).

**Table 1:** Patient characteristics

	LRR	DM	Controls	Significance
Number	32 *n*(%)	34 *n*(%)	49 *n*(%)	*P*-value
*Histology*				
IDC	30 (93)	29 (85)	43 (88)	0.1418
ILC	0	4 (12)	5 (10)	
Mixed IDC/ILC	0	1 (3)	1 (2)	
Adeno NOS	1 (3)	0	0	
Unknown	1 (3)	0	0	
*Hormone status*				
+	23 (72)	27 (79)	39 (79)	0.6800
−	9 (28)	7 (20)	10 (20)	
*Group stage*				
I	8 (25)	7 (20)	11 (23)	0.9767
IIA	17 (53)	18 (53)	22 (45)	
IIB	7 (22)	7 (20)	13 (26)	
IIIA	0	1 (3)	1 (2)	
Unknown	0	1(N0) (3)	1(N1) (2)	
*T stage*				
T1	19 (59)	12 (35)	31 (63)	0.1196
T2	12 (34)	20 (58)	16 (33)	
T3	1 (3)	1 (3)	1 (2)	
Unknown	0	1 (3)	1 (2)	
*N stage*				
N0	15 (47)	21 (62)	14 (29)	0.0168
N1	17 (53)	13 (38)	34 (69)	
*Margins*				
Negative	24 (75)	28 (82)	44 (90)	0.2883
Close (<2 mm)	4 (12)	5 (15)	3 (6)	
Unknown	4 (12)	1 (3)	2 (4)	
*Grade*				
1	0	0	4 (8)	0.2919
2	6 (19)	9 (26)	6 (12)	
3	24 (75)	22 (65)	33 (67)	
Unknown	2 (6)	3 (9)	6 (12)	
*Age*				
≤50	15 (47)	16 (47)	28 (57)	0.5607
>50	17 (53)	18 (53)	21 (30)	
*Adjuvant chemo*				
Yes	21 (66)	24 (71)	36 (73)	0.8248
No	11 (34)	10 (29)	11 (22)	
Unknown	0	0	2 (4)	
Adjuvant endocrine therapy (*n* = 87 ER+)	*n* = 23	*n* = 25	*n* = 39	0.2712
Yes	19 (83)	20 (80)	33 (85)	
No	4 (17)	4 (16)	2 (5)	
Unknown	0	1 (4)	4 (10)	

### DNA mutational analysis

Hybrid capture DNA sequencing was performed on DNA from primary samples on 57 patients (17 LRR, 15 DM, and 25 controls) and on matched primary tumor–normal tissue samples from 51 patients (15 LRR, 13 DM, and 23 controls). When genomic alterations were grouped by pathway the following were considered PI3K/Akt/mTOR pathway alterations: amplifications or mutation of *AKT1*, *AKT2*, *AKT3*; loss or mutation of *PTEN*; truncations or nonsense mutations in *TSC1* and *TSC2*; amplifications or mutations in PIK3CA, or *PIK3R1*. The following were considered mitogen-activated protein kinase (MAPK) alterations: amplifications/gains of *KRAS*, *BRAF*, or *RAF1*, or mutations/deletions of *NF1*. Sequencing was performed on one of two hybrid capture platforms (T200 and T200.V1) that performed whole exome sequencing on a panel of genes; the panels included 142 genes in common, listed in Supplementary Figure 1. Details of the mutations and allele frequencies are listed in Supplementary Table 1.

**Fig. 2:**
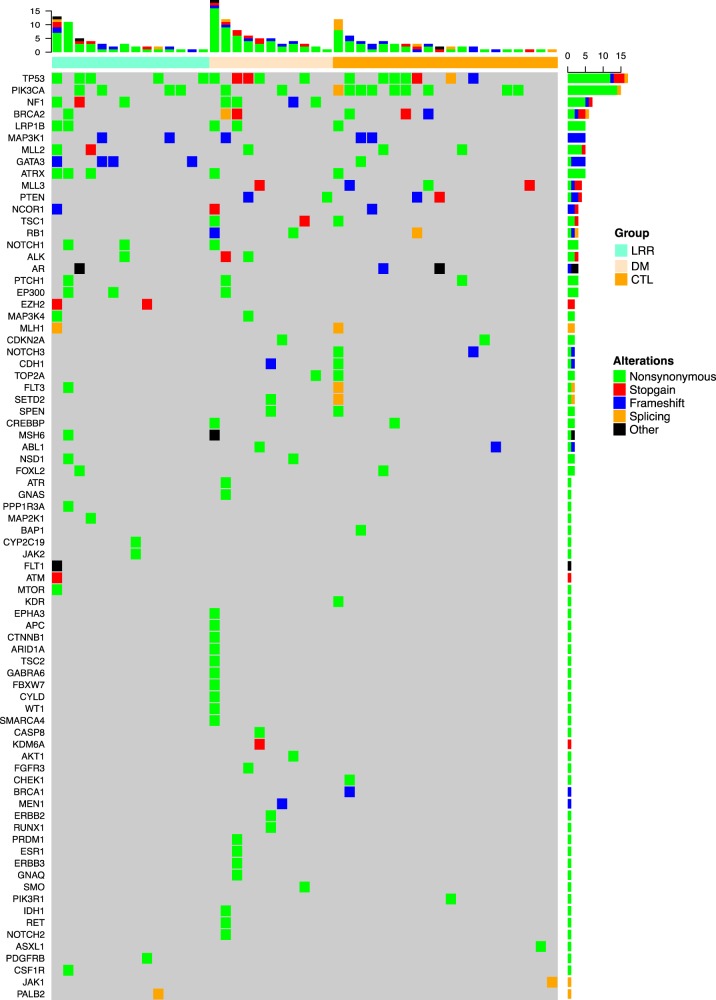
Somatic alterations in tumors from patients with loco-regional recurrence (LRR), distant metastasis (DM), and in controls without any type of recurrence

**Fig. 3:**
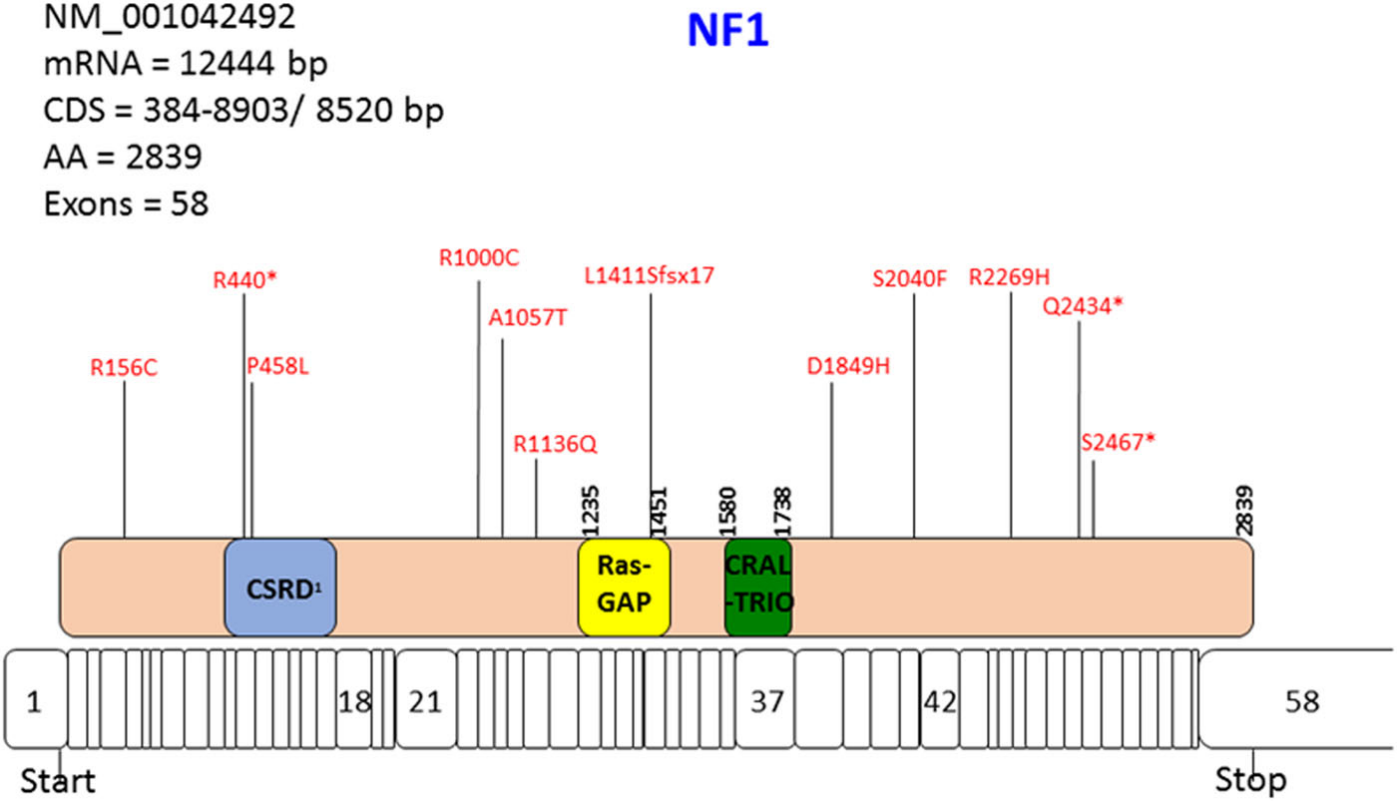
The somatic mutations of NF1 identified in the cohort

As expected *TP53* and *PIK3CA* were the most common mutations (Fig. 2). There were no significant differences in *TP53* mutation frequency in the primary tumors of patients who developed LRR or DM vs. control patients. There was a significantly higher proportion of *NF1* mutations among patients who developed LRR (24%) and in those who developed DM (27%) compared to patients who did not relapse (0%, *p* = 0.0213 and 0.0149, respectively). This was also significant when looking at any relapse vs. control (*p* = 0.0070). The somatic mutations noted in *NF1* are shown in Fig. 3; it should be noted that while some are likely deleterious as they are truncating or frameshift mutations, some of the *NF1* mutations were variants of unknown significance. Tumor blocks were not available for further analysis and it is unknown if the *NF1* mutation was sub-clonal in the primary tumor. The MAPK pathway was significantly mutated in both the LRR and DM patient samples compared to control samples (47%, 40%, 0% respectively; *p* < 0.0001 Table 2). This was also true when comparing LRR vs. control and DM vs. control, (*p* = 0.0002 and 0.0013, respectively). In contrast, taken together there was no significant difference in frequency of genomic alterations of the PI3K/AKT/mTOR pathways among the three groups.

**Table 2:** Comparison of genomic alterations in patients with loco-regional recurrence (LRR), distant metastatic disease (DM), and in the control group who did not develop any type of recurrence

	LRR *N* = 17 (%)	DM *N* = 15 (%)	Control *N* = 25 (%)	*P*-value
*TP53*	7 (41)	5 (33)	6 (24)	0.3906
*PIK3CA*	4 (23)	2 (13)	9 (36)	0.2252
*NF1*	4 (24)	4 (27)	0	0.0070
PI3K/Akt/mTOR pathway	13 (76)	11 (73)	15 (60)	0.2618
MAPK pathway	8 (47)	6 (40)	0	<0.0001

We also analyzed the frequency of genomic alterations by breast cancer subtype. *TP53* was significantly more frequently mutated in TNBC samples compared to HR+ samples. There was a trend toward more *PIK3CA* mutations in HR+ tumors (33% vs. 7%; *p* = 0.08). *TP53* mutations were significantly more likely in TNBC compared to HR+ (33% vs. 17%, *p* = 0.0096). There was no significant difference in *NF1* mutation rates by subtype (Supplementary Table 2). There were also no differences in the frequency of any genomic alterations when analyzing the cases when categorized by luminal A, luminal B, or triple negative subtypes.

**Fig. 4:**
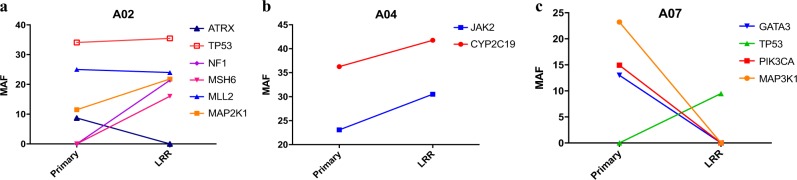
Comparison of somatic mutations in the original primary tumors and locally recurrent specimens. Patient A02: NF1 mutation was in the loco-regional recurrent (LRR) specimen but not reported in the primary tumor. There was one only read of this mutation upon review, and the primary tumor had lower coverage. It is unclear if NF1 mutation was acquired in the LRR or was preexisting and enriched in the LRR. Patient A04: Notable increase in Jak2 and CYP2C19 in the LRR compared to primary. Patient A07: There was a complete change in the genomic profile and the new tumor was also HER2 amplified. The results are more suggestive of a new primary rather than a true local recurrence

There were three patients with available primary and matching LRR tumor samples for DNA analysis (Fig. 4). One patient had additional mutations in the LRR, including gain of a *NF1* mutation (Fig. 4a), the second patient had concordant mutations (Fig. 4b), and the third had a complete mutation discordance and gain of HER2 amplification by FISH, suggestive of a new primary (Fig. 4c). Given the *NF1* mutation detected in the LRR and not the primary in sample A02, we re-reviewed the sequencing data on the primary tumor. On re-review there was one read of this mutation in the primary, and the primary had lower coverage. Thus it is possible that this *NF1* mutation was preexisting and was enriched in the LRR, rather than being acquired de novo.

### RNA

A total of 115 patients with RNA seq data were evaluated. Five samples were removed due to low mapping coverage leaving 110 patients for analysis (30 LRR, 33 DM, and 47 controls). PCA analysis did not show any correlation between expression profile and sample groups (e.g. stage at presentation). Differential gene expression (DEG) analysis also did not produce any significant DEGs when comparing the expression profile of primary tumors of control patients to those who developed LRR or DM. However; there are significant gene differences in 92 genes when comparing the patients with ER+ and ER− primary tumors (Supplementary Table 3). There were 13 recurrent LRR and 3 DM specimens, and there was no DEG when comparing the primary vs. recurrent samples. Five of these (4 LRR and 1 DM) samples were treated with adjuvant chemotherapy. There were also no significant differences between controls, LRR or DM samples, when the three groups were compared using the genes constituting the oncotype DX gene set, mammaprint, Prosigna PAM 50, and the wound response signature.^8–11^ Also, given low tumor cellularity may affect the RNA analysis, we also repeated the analysis only limiting the analysis to the samples with a mutant allele frequency of 20% or higher on DNA sequencing, and again no DEG were identified between the three groups.

### Ki-67, PTEN, and CC3 analysis

**Fig. 5:**
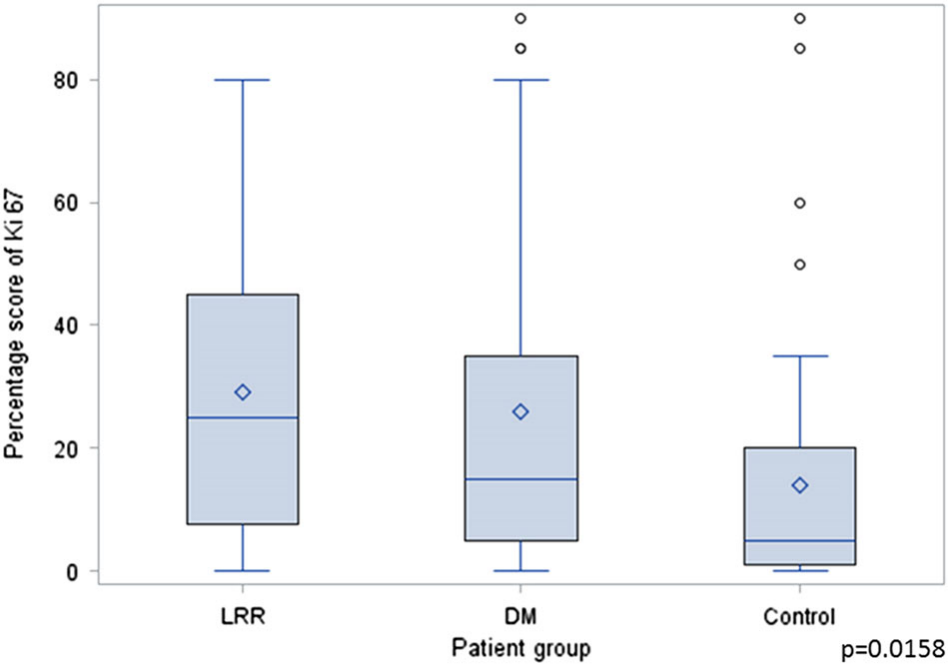
Comparison of Ki 67 expression in the primary tumors of patients who developed a recurrence, loco-regional recurrence (LRR) or distant metastasis (DM), to control patients who did not develop any type of recurrence. Ki 67 expression is significantly elevated in patients with either a LRR or DM compared to controls

The mean proportion of the Ki-67 positive cells was 29.2% for patients who developed LRR, 26.0% for patients who developed a DM, and 14.0% for controls. When comparing the three groups, the Ki-67 proliferation index was significantly different between groups by ANOVA; *p* = 0.0158 (Fig. 5). Ki-67 was significantly higher in any recurrence vs. control, LRR vs. control and DM vs. control; *p* = 0.0045, *p* = 0.0013, and *p* = 0.0238, respectively.

PTEN was negative in the 13 patients that developed LRR (41%), the 12 that developed DM (35%) and in 21 control patients (44%). The mean proportion of the CC3-positive cells was 2.7% for patients with LRR, 3.9% for patients with DM, and 3.8% for primary tumors of control patients. There was no difference when comparing any recurrence vs. control, LRR vs. control, or DM vs. control in the frequency of PTEN loss or apoptosis as determined by CC3.

HR+ patients were, however, significantly more likely to have a positive PTEN, lower Ki-67 expression and lower CC3 expression compared to HR− patients; *p* = 0.0004, *p* < 0.0001, and *p* < 0.0001, respectively. Amongst the HR+ patients, Ki-67 mean scores were significantly elevated when comparing LRR (32.16%) to control and DM (25.46%) to control (13.9%), *p* = 0.0087 and *p* = 0.0293, respectively. PTEN and CC3 were not significantly different between the groups. There was no association between *NF1* and an elevated Ki-67; *p*-value = 0.1835 (Supplementary Figure 2). The IHC results and tumor subtype are listed for the patient cohorts in Supplementary Table 4.

## Discussion

Great controversy exists regarding the need for radiation treatment for post-mastectomy patients with one to three positive lymph nodes. Although randomized studies have demonstrated a survival advantage for post-mastectomy radiation, there are concerns and criticisms surrounding the surgical techniques and now outdated systemic therapy.^12–14^ Retrospective studies have shown mixed results, thus the magnitude of benefit with radiation remains elusive. Recent institutional studies have suggested the LRR in patients with 1–3 positive nodes may be quite low in the modern era.^4–6^ Thus, it is crucial to identify patients both with sufficiently low and high risks of a recurrence to avoid under or over-treatment. The principal goal of our study was to determine whether molecular profiling could assist in predicting post-mastectomy loco-regional recurrence. We designed this study to compare patients with LRR to patients with DM and controls without any recurrence.

First, it should be noted that the identification of even a small cohort of patients with LRR after mastectomy in the setting of 0–3 positive nodes for this study necessitated a multi-center study. This is in part due to many patients not being eligible due to having received neoadjuvant systemic therapy, post-mastectomy radiation therapy, or both. However, we believe this reflects the fact that the natural history of patients with N1 disease has evolved with modern adjuvant therapy and imaging, and that isolated LRR may in fact be an infrequent occurrence. Indeed, McBride et al. reported that unlike patients with T1 or T2 cancer with 1–3 positive nodes treated with mastectomy and systemic therapy in an earlier era (1978–1997), patients treated in a later time period (2000–2007) did not appear to benefit from PMRT.^15^ This further emphasizes that LRR rates after mastectomy in N1 patients, treated with contemporary surgical and systemic therapy, may be low, and identifying patients at higher risk of recurrence would be essential to avoid over-treating the entire N1 population.

Past efforts to identify molecular predictors of LRR include work by Cheng et al. who analyzed 94 patients treated with mastectomy without radiation therapy and found two sets of genes (one with 258 genes and the other with 34 genes) to be predictive of LRR.^16^ The Netherlands group explored gene expression profiles in 161 patients treated with breast conservation, 17 with a subsequent LRR,^11^ and found that the wound response signature was significantly associated with occurrence of LRR. A re-analysis, hindered by small numbers, failed to find a significant gene profile that could predict LRR.^17^ An expansion of this cohort to 165 patients, 56 with a LRR, found that genes associated with cell proliferation were expressed at higher levels in tumors from patients with a LRR.^18^ LRR were more likely to occur in tumors that were luminal B or HER2-like. The 70-gene signature and the chromosomal instability signature were also found to be significant predictors of LRR. However, the wound response signature’s ability to predict LRR was not confirmed in this larger cohort. The National Surgical Adjuvant Breast and Bowel Project (NSABP) analyzed the Oncotype-DX 21-gene recurrence score (RS) assay and its ability to quantify the risk of LRR in patients with node-negative, ER+ breast cancer from two NSABP trials, B14 and B20.^19^ Eight-hundred and ninety-five patients were treated with tamoxifen, 355 treated with placebo only, and 424 treated with chemotherapy and tamoxifen. The RS was significantly associated with local recurrence for all subsets of patients. The association between RS and LRR was also assessed in node-positive, ER-positive patients treated with adjuvant chemotherapy plus tamoxifen in NSABP B-28.^20^ RS was a statistically significant predictor of LRR in univariate analyses (10-year cumulative incidence of LRR = 3.3%, 7.2%, and 12.2% for low, intermediate, and high RS, respectively, *p* < 0.001). There was a statistically nonsignificant trend between RS and risk of LRR in patients with one to three positive nodes. The 10-year cumulative incidence of LRR was 3.2%, 5.1%, and 7.9% for low, intermediate, and high RS, respectively (*p* = 0.12). For patients receiving mastectomy with one to three positive nodes, rates of local recurrence were low (local recurrence: 1.6%, 1.7%, and 2.6%, for low, intermediate, and high RS, respectively) as were rate of regional recurrences (0.8%, 2.4%, and 3.4% for low, intermediate, and high RS, respectively).

In our highly selected patient population, all of whom underwent mastectomy without radiation, the RNA profile could not predict for either LRR or DM when analyzing the primary tumors. We also evaluated the previously published molecular signatures, which have been widely used to predict the development of recurrence, and did not find significant association with these signatures and risk of LRR or DM. It is possible that we could not detect such a signature due to small sample size. However, it may also been attributable to suboptimal RNA preservation (due to differing fixation methods, aged blocks, etc.) limiting RNA seq quality. We accessed the quality of RNA seq data and excluded samples with low-quality aggressively. Another concern is patient/tumor and treatment heterogeneity in our series. Future studies should focus on a single biologic subtype so any molecular signal would not be blunted by tumor heterogeneity.

Germline mutations in *NF1* are associated with neurofibromatosis type 1 an autosomal dominantly inherited tumor predisposition syndrome. Although the impact of *NF1* on breast cancer risk has been controversial, it has been reported that patients with *NF1* have an increased risk of developing breast cancer compared to the general population; women with *NF1* <50 years of age have a 4–5 fold increased risk of developing breast cancer and have an increased proportionate mortality ratio.^21–23^ Somatic *NF1* mutations may be a driver in cancers occurring at a number of different sites: breast, colorectum, urothelium, lung, ovary, skin, brain, and neuroendocrine tissues, as well as leukemia.^24^ Our data suggest that *NF1* can also be a somatic mutation in breast cancer, conferring an increased risk of recurrence. Our results need validation as the frequency of *NF1* mutations in our study, both in patients with LRR (24%) and in those with DM (27%) is surprisingly high, compared to the 2–6% frequency of *NF1* mutations observed in the TCGA and other genomic profiling series.^25,26^ However, the higher rate of *NF1* mutations in patients that relapsed as well as the gain or enrichment of a *NF1* mutation in a patient with LRR both suggest that *NF1* mutations may play a role in poor prognosis. Notably, *NF1* mutations may activate both PI3K and MAPK signaling and it is thought different *NF1*-mutant tumors may be more or less dependent on different ras effectors. We classified *NF1* mutations as a MAPK alteration, consistent with recent breast cancer literature, and because of the greater interest in targeting *NF1* germline-mutant tumors with MAPK targeting *NF1*.^27,28^ MAPK pathway is involved in many cellular processes including cell proliferation, differentiation, and transcriptional regulation. Studies evaluating MAPK genomic alterations in breast cancer are few in number; however, activation of MAPK signaling can confer resistance to therapy resulting in poorer outcomes, and MAPK pathway activation may promote immune evasion.^29–31^ This is in keeping with our findings that MAPK pathway mutations are significantly more common in primary tumor samples from patients who recurred. Further study is needed to determine whether *NF1* mutations and MAPK activation confers a worse prognosis by increasing LRR and DM potential, or whether they confer resistance to adjuvant chemotherapy and endocrine therapy. It is also interesting to note that *NF1* was not associated with an elevated Ki-67 in our cohort. This could be, in part, due to the small number of total *NF1* cases (*n* = 8); however, the Ki-67 ranged from 1% to 75% in our study so it could be that *NF1* mutations are independent of Ki-67.

Increased cell proliferation and/or decrease in apoptosis are indicators of more aggressive tumor biology. Many molecular marker studies have identified genes associated with proliferation to be predictive of outcome.^32,33^ Thus, we hypothesized that Ki-67 alone or in conjunction with the apoptosis marker CC3 may correlate with a higher risk for LRR. In our study, while there was no correlation between CC3 and outcome, high Ki-67 did correlate with the development of a LRR and DM, and a decreased expression was significantly associated with HR+ tumors. This raises the possibility of using Ki-67, a low cost assay, in our clinical armamentarium for therapeutic decision-making. Further research is needed, however, as Ki-67 has operator-dependent reproducibility as a significant limitation and the definition of what constitutes a “high Ki-67” varies across institutions.^34^ It is best taken into context with other patient and tumor prognostics factors as no single parameter will likely accurately predict the risk of recurrence.

Our study has several limitations. First, this was a case-cohort study by design, but ultimately sample size was limited both due to difficulty in case identification, as well as inadequacy of samples due to their poor quality. Second, although we initially planned to have a case-control design, with each LRR or DM case matched to a control, this ended up being challenging. Due to tumor cellularity and clinical ineligibility upon review, many of the “matches” failed. Ultimately when control patients with adequate tumor samples were compared to those with LRR and DM, they were overall not significantly different in patient and tumor characteristics (Table 1), but interestingly more of the control patients had N1 (vs. N0) disease. Third, the RNA analysis may have been less informative due to modest cellularity, differences in tissue fixation, age of tumor samples as well as low sample number posed significant challenges. Fourth, although we tried to match treatments between cases and controls, overall patients were treated with a variety of systemic treatments, and we included both HR+ and HR− HER2 negative tumors in our analysis, adding additional heterogeneity. HER2 positive disease was a specific exclusion criterion in our population. Fifth, the time period for study was selected to reflect current systemic therapy practice including taxanes and aromatase inhibitors, however, over the past few years surgical practice is also changing, with a decreased use of completion axillary lymph node dissection. However, this further highlights the need for molecular predictors of patients at high risk of LRR. Finally, here we focused on identifying determinants of LRR and DM. However, we have not yet addressed whether patients with high risk features will be responsive to intervention with PMRT or more aggressive or targeted systemic therapy.

## Conclusions

While our sample size was small it did provide insight into possible drivers of recurrence. *NF1* somatic mutations specifically and MAPK pathway alterations overall are worthy of further exploration as potential prognostic markers as these were significantly more likely in patient tumor samples which had recurred. Further study is needed for validation of these findings and to determine whether biomarker-driven treatment stratification can reduce the need for radiation while maintaining excellent locoregional and distant recurrence rates.

## Methods

This is a multicenter retrospective study of patients who underwent mastectomy at a participating Translational Breast Cancer Research Consortium (TBCRC) institution. Study centers included Memorial Sloan Kettering Cancer Center, University of Pittsburgh, Duke University, Dana Farber Cancer Institute, University of North Carolina at Chapel Hill, Georgetown University, University of Michigan at Ann Arbor, Vanderbilt University, University of Alabama at Birmingham and MD Anderson Cancer Center and IRB approval and waiver of consent, due to the retrospective nature of the study, was provided at each institution. IRB approval numbers are as follows: UNC IRB #13-3365; Vanderbilt IRB #131361; MSKCC IRB #IRB WA0214-13 and HBS2013041; MD Anderson IRB #PA11-0416; Michigan IRB #HUM00076548; DFCI IRB #13-391; Georgetown IRB #2014-0577; Duke IRB #Pro00049761; Pittsburgh IRB #PRO13050254/0502025; UAB IRB#130903001 and 130807006.

Eligible patients included those patients who underwent mastectomy without radiation for T stage 1–3 and N stage 0–1 breast cancer, and for whom archived tissues and outcome data were available. Patients with any recurrence, loco-regional (LRR) or with distant metastasis (DM), were treated from 1997 to present time as this was felt to represent an era when treatment recommendations would be the most uniform and consistent. Patients with LRR could have a subsequent DM as long as this DM was diagnosed at least 3 months after the LRR. Patients who developed LRR after developing distant metastases were placed in the DM group. Control patients were treated from 1997 to 2007 to allow at least 5 years follow-up with a disease-free interval. Controls were matched to cases for age, ER/PR status, chemotherapy and endocrine therapy regimens and year of diagnosis. All patients were treated with an upfront mastectomy followed by systemic therapy if indicated, which included chemotherapy, endocrine therapy, or both. Patients were ineligible if they received post-mastectomy radiation therapy, neoadjuvant chemotherapy, had a T4 primary tumor, N2 nodal disease, were HER2 positive, had positive margins after mastectomy, had fewer than 10 lymph nodes retrieved at axillary lymph node dissection, or had inadequate follow-up (<5 years) if a control patient. If specimens were available from the recurrent tumors, these were also collected for exploratory analysis. The sample size was decided by the feasibility to collect breast cancer tissue samples from patients with LRR, DM, and patients without any recurrence (controls). With a size of 50 cases with LRR, 50 cases with DM, and matched controls, we would expect detection of a modest to large molecular expression difference with a reasonable false discovery rate of 30% or less.

### DNA sequencing

One 5u slide for H&E and 4 *adjacent* 10u slides were sent for each case. Tumor samples were macrodissected and genomic DNA was extracted using QIAamp DNA FFPE Micro Kit (Qiagen) and quantified by Qubit (Invitrogen). Normal tissue was used as a normal germline control. NGS was performed on tumor and normal DNA as previously described.^35,36^ Sequencing was performed on one of two hybrid capture platforms (T200 and T200.V1) that performed whole exome sequencing a panel of genes; the panels included 142 genes in common, listed in Supplementary Figure 1. The DNA analysis in this manuscript was focused on these 142 genes. The analysis was performed as previously described.^36^ Eleven patients were excluded due to samples showing low depth of coverage.

### RNA sequencing

For cases with limited yield, the RNA seq was prioritized over DNA seq analysis. After isolation, RNA was quantified by Picogreen (Invitrogen) and quality was assessed using the 2200 Tapestation (Agilent). RNA from each sample (10–100 ng) was converted in double stranded cDNA using Ovation RNA-Seq System V2 kit from Nugen. cDNA was sheared by sonication with the following conditions: Peak Incident Power 175, Duty Cycle 20%, Intensity 5, Cycles per Burst 200, and 120 s using Covaris E220 instrument (Covaris). The sheared DNA proceeded to library prep using KAPA library prep hyper kit (KAPA) following the “with beads” manufacturer protocol. At the end of the library prep, samples were analyzed on TapeStation to verify correct fragment size and to ensure the absence of extra bands. Samples were quantified using KAPA qPCR quantification kit. DNA was pooled for capture (2–6 samples per pool). We used whole exome biotin-labeled probes from Roche Nimblegen (V3) and followed manufacture’s protocol for the capture step. The quality of each captured sample was analyzed on TapeStation using the DNA High Sensitivity kit and the enrichment was accessed by qPCR using specific primers designed by Roche Nimblegen. The cutoff for the enrichment was 50-fold minimum.

The captured libraries were sequenced on a HiSeq 2500 (Illumina Inc., San Diego, CA, USA) on a version 3 TruSeq paired end flowcell according to manufacturer’s instructions at a cluster density between 700 and 1000 K clusters/mm^2^. Sequencing was performed on a HiSeq 2000 for 2 × 100 paired end reads with a 7 nt read for indexes using Cycle Sequencing v3 reagents (Illumina). The resulting BCL files containing the sequence data were converted into “.fastq.gz” files and individual libraries within the samples were demultiplexed using CASAVA 1.8.2 with no mismatches. All regions were covered by >20 reads.

Data analysis was performed using a comprehensive in-house RNA Seq pipeline. We used tophat to align paired-end reads to the hg19 version of the reference genome, htseq-count, and bedtools to obtain expression counts of genes and exons. Genetic variants were called using GATK unified genotype. Quality of raw and aligned reads was assessed using FastQC and RSEQC. We used QualiMap to evaluate alignment results across samples and used quality of RNA Seq samples to assess quality of mutations called from corresponding DNA samples. Samples with low depth of coverage in RNA were found to be associated with high numbers of somatic mutations and were excluded from further analysis.

### Immunohistochemistry

Immunostaining was performed as previously described.^37^ The following antibodies were used: Ki67 (Dako #M7240,) and PTEN (Dako #M3627) and CC3 (Cell Signaling #DC8). Negative control slides, without primary antibody, were included for each sample. Cases in which internal control staining was not observed or equivocal were considered un-interpretable. A binary scoring system was used for interpretation. A negative sample was defined as no staining in ≥90% of the tumor cells. A positive sample was defined as >10% of tumor cells staining. Ki-67 was scored as % positive cells. Apoptosis was assessed by CC3.

### Statistical analysis

#### DNA and RNA analysis

The frequency of common alterations on NGS was compared with Fisher’s exact test analysis. When genomic alterations were grouped by pathway the following were considered PI3K/Akt/mTOR pathway alterations: amplifications or mutation of *AKT1*, *AKT2*, *AKT3*; loss or mutation of *PTEN*; truncations or nonsense mutations in *TSC1* and *TSC2*; amplifications or mutations in PIK3CA, or *PIK3R1*. The following were considered MAPK alterations: amplifications/gains of *KRAS*, *BRAF*, or *RAF1*, or mutations/deletions of *NF1*.

Expression of each gene from mRNA-Seq was treated as an explanatory variable. We analyzed expression data for presence of clusters based on DEG by using available methods with the R statistical software package (http://cran.r-project.org) using a variety of unsupervised clustering methods (including hierarchical clustering, K-means, and gene shaving) to classify the samples into statistically similar groups. We retained clusters that a) are robust to bootstrap re-sampling; b) are found by multiple clustering algorithms; and c) contain ≥10% of the samples.

#### PTEN

*PTEN* mutations were assessed as dichotomous variables. Fisher’s exact test or Chi-Square statistics were used to determine whether *PTEN* alterations of the primary tumor correlate with development of LRR, DM, or both. We used NGS and IHC for *PTEN* loss. Logistic regression analysis was further used to evaluate if *PTEN* mutations were predictors of recurrence.

#### Ki 67 and CC3

The percentage of cells that express Ki-67 and CC3 were analyzed as a continuous variable. Percentage of Ki-67 and CC3-positive cells from patients who developed a LRR, DM, or both were compared to control patients with a nonparametric methods.

## Electronic supplementary material

Supplementary Figures and Tables

## Data Availability

All somatic mutation data is available in Supplementary Table S1, including the mutations and allele frequencies in our patient dataset. Supplementary Table S4 contains the tumor subtype and IHC data for our patient dataset. Patient samples are the property of the individual institutions from which they were obtained. The DNA mutation calls discussed in this publication have been deposited in EMBL-EBI’s European Variation Archive (Project: PRJEB28702, Analyses: ERZ724019). The RNA Sequencing data are accessible through GEO Series accession number GSE119937.

## Electronic supplementary material

**Supplementary information** accompanies the paper on the *npj Breast Cancer* website (10.1038/s41523-018-0089-z).
