# Supplementary material for: Molecular determinants of post-mastectomy breast cancer recurrence
Source: NPJ Breast Cancer. 2018 Oct 12;4:34. doi: 10.1038/s41523-018-0089-z (PMC6185974; doi:10.1038/s41523-018-0089-z)
Supplement: Supplementary file 1 — Supplementary Figures and Tables [file 41523_2018_89_MOESM1_ESM.pdf]

ABL1	CEBPA	FGFR3	MAP2K1	PALB2	SOS1	CRIPAK	LRP1	ZNF238
ACVR1B	CHEK1	FGFR4	MAP2K4	PAX5	SPEN	CSMD1	LRP2	ZNF536
AKT1	CHEK2	FLT1	MAP3K1	PBRM1	SPOP	CSMD2	MDN1	
ALK	CREBBP	FLT3	MAP3K4	PDGFRA	STK11	CSMD3	MECOM	
APC	CSF1R	FLT4	MEN1	PDGFRB	SYK	DDR1	NAV3	
AR	CTNNB1	FOXL2	MET	PIK3CA	TET2	ELN	NFKB2	
ARAF	CYLD	GABRA6	MITF	PIK3CG	TGFb1	EML4	PAPPA2	
ARID1A	CYP2C19	GATA1	MLH1	PIK3R1	TGFBR2	ETV5	PCDH15	
ASXL1	DAXX	GATA3	MLL2	PPP1R3A	TNFAIP3	FAM135B	PCLO	
ATM	DDR2	GNA11	MLL3	PPP2R1A	TOP1	FAT3	PIKFYVE	
ATR	DNMT3A	GNAQ	MPL	PRDM1	TOP2A	FLG	PKHD1	
ATRX	EGFR	GNAS	MSH2	PTCH1	TP53	GABRB3	PKHD1L1	
ARUKA	EP300	HNF1A	MSH6	PTEN	TSC1	HDAC9	PPP2R4	
ARUKB	EPHA3	HRAS	MTOR	PTPN11	TSC2	HEATR7B2	PRSS1	
BAP1	ERBB2	IDH1	MYD88	RAD51	TSHR	HGF	PTK2	
BRAF	ERBB3	IDH2	NCOR1	RAF1	VHL	HMCN1	RELN	
BRCA1	ERCC3	IGF1R	NF1	RB1	WT1	HNF1B	RIMS2	
BRCA2	ERCC4	JAK1	NF2	RET	ADAMTS12	HYDIN	RNF213	
CARD11	ERCC5	JAK2	NOTCH1	RUNX1	AKAP3	IKZF1	RYR2	
CASP8	ESR1	JAK3	NOTCH2	RUNX1T1	BAI3	IL6R	SPTA1	
CBL	EZH2	KDM6A	NOTCH3	SETD2	CD19	IRS1	SYNE1	
CDH1	FAM123B	KDR	NOTCH4	SMAD4	CDH10	ITGA4	SYNE2	
CDK1	FBXW7	KIT	NPM1	SMARCA4	CDH11	KCNB2	TBC1D4	
CDK6	FGFR1	KRAS	NRAS	SMARCB1	COL14A1	LAMA1	USH2A	
CDKN2A	FGFR2	LRP1B	NSD1	SMO	CPAMD8	LPHN3	WHSC1	

Figure S1: Genes tested using the T200 gene platform. Eighty of these genes are therapeutically actionable based on the potential to be targeted with approved or investigational therapies.

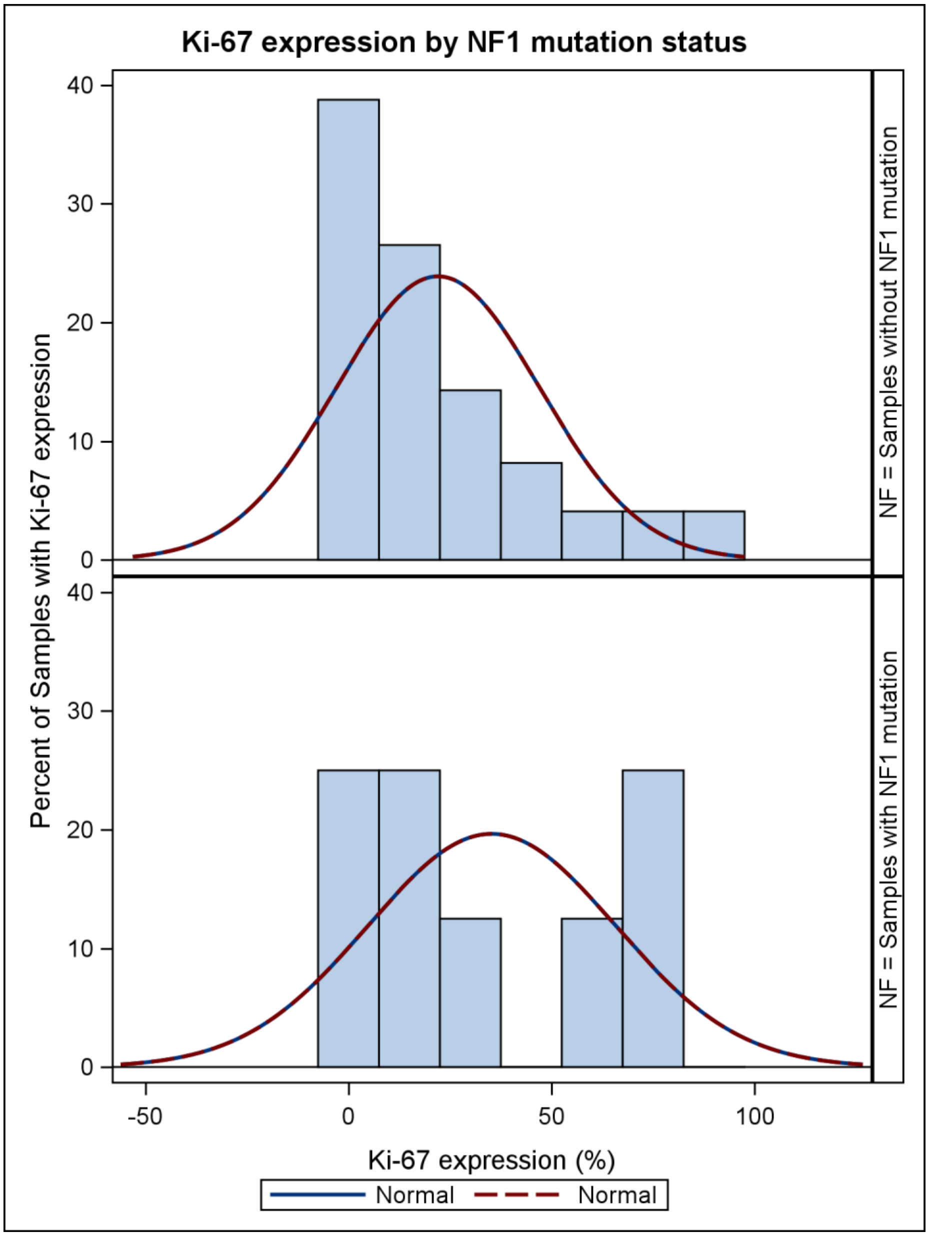

Figure S2: Comparison of Ki 67 expression in patients that do or do not have a *NF1* mutation. There is no significant difference in Ki 67 expression based on *NF1* mutation status; p-value = 0.1835.

Table S1: DNA mutations and allele frequencies in patients with locoregional recurrence (LRR), distant metastasis (DM) or controls (CTL).

Patient ID	group	Type	mutation	HGNC	Allele Frequency	Coverage	Consequence
1	DM	Primary_DNA	9_21971185_G/A	CDKN2A	20.55%	1090	non_synonymous_codon
1	DM	Primary_DNA	3_178936091_G/A	PIK3CA	6.68%	577	non_synonymous_codon
1	DM	Primary_DNA	11_64572575_-/GGGCAGT	MEN1	2.33%	946	frameshift_variant
1	DM	Primary_DNA	3_178938934_G/A	PIK3CA	11.23%	1657	non_synonymous_codon
2	CTL	Primary_DNA	X_66765243_GCA/-	AR	6.23%	289	inframe_codon_loss
2	CTL	Primary_DNA	10_89720789_G/T	PTEN	28.16%	4063	stop_gained
4	CTL	Primary_DNA	X_66765384_C/-	AR	18.42%	152	frameshift_variant
4	CTL	Primary_DNA	17_7577120_G/A	TP53	30%	150	non_synonymous_codon
4	CTL	Primary_DNA	12_49444379_C/A	MLL2	6.15%	65	non_synonymous_codon
4	CTL	Primary_DNA	3_138664622_G/A	FOXL2	30.77%	13	non_synonymous_codon
6	DM	Primary_DNA	17_37880220_T/C	ERBB2	63.76%	5176	non_synonymous_codon
6	DM	Primary_DNA	16_68849632_A/-	CDH1	49.25%	1330	frameshift_variant
6	DM	Primary_DNA	3_47098913_C/T	SETD2	27.96%	1302	non_synonymous_codon
6	DM	Primary_DNA	1_16259654_G/A	SPEN	31.18%	1892	non_synonymous_codon
6	DM	Primary_DNA	21_36421143_G/A	RUNX1	17.18%	3190	non_synonymous_codon
8	DM	Primary_DNA	12_56495339_C/A	ERBB3	5.72%	367	non_synonymous_codon
8	DM	Primary_DNA	9_80336264_A/T	GNAQ	38.30%	1295	non_synonymous_codon
8	DM	Primary_DNA	13_32914890_C/G	BRCA2	54.85%	392	stop_gained
8	DM	Primary_DNA	17_7578257_G/A	TP53	37.05%	1039	non_synonymous_codon
8	DM	Primary_DNA	2_141055429_C/A	LRP1B	61.46%	698	non_synonymous_codon
8	DM	Primary_DNA	17_29654793_G/C	NF1	61.81%	542	non_synonymous_codon
8	DM	Primary_DNA	17_7577022_C/T	TP53	77.81%	951	stop_gained
8	DM	Primary_DNA	6_152265535_G/A	ESR1	8.20%	61	non_synonymous_codon
8	DM	Primary_	6_106552939_C/T	PRDM1	33.42%	1155	non_synonymous_codon

		DNA					
10	LRR	Primary_DNA	17_7577082_G/A	TP53	35.83%	1169	non_synonymous_codon
10	LRR	Primary_DNA	15_66782854_C/G	MAP2K1	21.81%	541	non_synonymous_codon
10	LRR	Primary_DNA	12_49423006_G/T	MLL2	23.98%	392	stop_gained
10	LRR	Primary_DNA	X_76849197_C/A	ATRX	8.72%	172	non_synonymous_codon
10	LRR	Recurrent_DNA	12_49423006_G/T	MLL2	23.98%	392	stop_gained
10	LRR	Recurrent_DNA	17_7577082_G/A	TP53	35.83%	1169	non_synonymous_codon
10	LRR	Recurrent_DNA	2_48026064_C/G	MSH6	16%	600	non_synonymous_codon
10	LRR	Recurrent_DNA	15_66782854_C/G	MAP2K1	21.81%	541	non_synonymous_codon
10	LRR	Recurrent_DNA	17_29663463_C/T	NF1	21.37%	262	non_synonymous_codon
12	LRR	Recurrent_DNA	11_64577506_GCCG/-	MEN1	6.39%	219	frameshift_variant
12	LRR	Recurrent_DNA	7_151945625_T/-	MLL3	21.78%	404	frameshift_variant
12	LRR	Recurrent_DNA	11_64577511_GC/-	MEN1	6.67%	210	frameshift_variant
13	CTL	Primary_DNA	3_178952085_A/G	PIK3CA	20.96%	225	non_synonymous_codon
13	CTL	Primary_DNA	3_52436943_G/A	BAP1	7.81%	64	non_synonymous_codon
13	CTL	Primary_DNA	10_8106058_T/G	GATA3	35.98%	895	non_synonymous_codon
13	CTL	Primary_DNA	5_56176933_-/G	MAP3K1	41.35%	399	frameshift_variant
13	CTL	Primary_DNA	3_178938934_G/A	PIK3CA	11.23%	1657	non_synonymous_codon
14	LRR	Primary_DNA	10_96541696_C/T	CYP2C19	41.76%	273	non_synonymous_codon
14	LRR	Primary_DNA	9_5044446_T/C	JAK2	30.51%	272	non_synonymous_codon
14	LRR	Recurrent_DNA	10_96541696_C/T	CYP2C19	41.76%	273	non_synonymous_codon
14	LRR	Recurrent_DNA	9_5044446_T/C	JAK2	30.51%	272	non_synonymous_codon
16	LRR	Primary_DNA	17_29665144_G/A	NF1	5.26%	76	non_synonymous_codon
16	LRR	Primary_DNA	7_148534068_C/T	EZH2	4.27%	117	stop_gained NMD_transcript_variant
16	LRR	Primary_DNA	11_108160338_C/T	ATM	8%	50	stop_gained
16	LRR	Primary_DNA	2_141533695_G/A	LRP1B	6.35%	63	non_synonymous_codon
16	LRR	Primary_	13_28942717_CAT	FLT1	8.47%	59	stop_retained_variant

		DNA	/-				inframe_codon_loss
16	LRR	Primary_ DNA	1_11273601_C/T	MTOR	5.26%	95	non_synonymous_codon
16	LRR	Primary_ DNA	17_15961884_G/A	NCOR1	4.35%	92	non_synonymous_codon
16	LRR	Primary_ DNA	17_15971417_A/-	NCOR1	42.31%	26	frameshift_variant
16	LRR	Primary_ DNA	10_8115874_-/C	GATA3	30.41%	148	frameshift_variant
16	LRR	Primary_ DNA	6_161471001_G/A	MAP3K4	5.15%	97	non_synonymous_codon
16	LRR	Primary_ DNA	3_37056036_G/A	MLH1	5.21%	96	splice_donor_variant
16	LRR	Primary_ DNA	X_76891412_G/A	ATRX	6.90%	58	non_synonymous_codon
16	LRR	Primary_ DNA	17_7578534_G/T	TP53	56.41%	54	non_synonymous_codon
16	LRR	Primary_ DNA	12_49445773_C/T	MLL2	5.56%	72	non_synonymous_codon
17	CTL	Primary_ DNA	9_21971036_G/A	CDKN2A	13.20%	341	non_synonymous_codon
18	LRR	Recurren t_DNA	2_29451809_G/A	ALK	11.80%	373	non_synonymous_codon
18	LRR	Recurren t_DNA	10_89690810_G/T	PTEN	11.85%	135	stop_gained
20	LRR	Primary_ DNA	5_56171018_G/-	MAP3K1	23.21%	168	frameshift_variant
20	LRR	Primary_ DNA	10_8111554_-/TT	GATA3	13%	200	frameshift_variant
20	LRR	Primary_ DNA	3_178936091_G/A	PIK3CA	6.68%	577	non_synonymous_codon
20	LRR	Recurren t_DNA	17_7578550_C/T	TP53	8.23%	521	non_synonymous_codon
21	CTL	Primary_ DNA	17_38568058_C/T	TOP2A	7.95%	88	non_synonymous_codon
21	CTL	Primary_ DNA	3_178952085_A/G	PIK3CA	20.96%	225	non_synonymous_codon
21	CTL	Primary_ DNA	2_141597638_G/A	LRP1B	8.33%	48	non_synonymous_codon
21	CTL	Primary_ DNA	13_28609631_G/A	FLT3	7.69%	52	splice_donor_variant
21	CTL	Primary_ DNA	2_141274476_C/T	LRP1B	6.30%	127	non_synonymous_codon
21	CTL	Primary_ DNA	3_178866392_G/A	PIK3CA	5.68%	88	splice_donor_variant
21	CTL	Primary_ DNA	2_141598486_G/A	LRP1B	6.78%	59	splice_donor_variant
21	CTL	Primary_ DNA	X_76938701_G/A	ATRX	15.91%	176	non_synonymous_codon
21	CTL	Primary_ DNA	3_37038105_G/T	MLH1	8.89%	45	splice_region_variant intron_variant
21	CTL	Primary_	9_135798764_G/A	TSC1	4.69%	128	non_synonymous_codon

		DNA					
21	CTL	Primary_ DNA	16_68862106_C/T	CDH1	4.52%	155	non_synonymous_codon
21	CTL	Primary_ DNA	2_141986859_G/A	LRP1B	5.13%	78	non_synonymous_codon
21	CTL	Primary_ DNA	19_15272099_C/T	NOTCH3	11.43%	35	non_synonymous_codon
21	CTL	Primary_ DNA	4_55972922_G/A	KDR	6.35%	63	non_synonymous_codon
21	CTL	Primary_ DNA	3_47147611_G/A	SETD2	7.69%	52	splice_acceptor_variant
21	CTL	Primary_ DNA	1_16256585_G/A	SPEN	3.85%	130	non_synonymous_codon
22	DM	Primary_ DNA	3_41280636_C/T	CTNNB1	5.06%	79	non_synonymous_codon
22	DM	Primary_ DNA	3_89259335_G/A	EPHA3	7.55%	53	non_synonymous_codon
22	DM	Primary_ DNA	5_161114522_T/C	GABRA6	5.48%	73	non_synonymous_codon
22	DM	Primary_ DNA	13_48955531_T/-	RB1	84.83%	178	frameshift_variant
22	DM	Primary_ DNA	11_32414263_C/G	WT1	49.41%	170	non_synonymous_codon
22	DM	Primary_ DNA	19_11129650_C/G	SMARC A4	38.28%	290	non_synonymous_codon
22	DM	Primary_ DNA	4_153247288_G/T	FBXW7	6.10%	118	non_synonymous_codon
22	DM	Primary_ DNA	16_2131629_C/T	TSC2	5.61%	107	non_synonymous_codon
22	DM	Primary_ DNA	X_76953080_G/A	ATRX	5.56%	72	non_synonymous_codon
22	DM	Primary_ DNA	2_141093221_G/A	LRP1B	5.48%	73	non_synonymous_codon
22	DM	Primary_ DNA	5_112177106_G/A	APC	5.80%	69	non_synonymous_codon
22	DM	Primary_ DNA	9_139391826_C/T	NOTCH1	5.06%	79	non_synonymous_codon
22	DM	Primary_ DNA	17_7578212_C/T	TP53	87.60%	176	stop_gained
22	DM	Primary_ DNA	2_48025764_C/T	MSH6	6.67%	75	NMD_transcript_variant non_synonymous_codon
22	DM	Primary_ DNA	9_135798851_C/A	TSC1	4.35%	92	non_synonymous_codon
22	DM	Primary_ DNA	16_3808953_C/T	CREBBP	5.63%	71	non_synonymous_codon
22	DM	Primary_ DNA	1_27023724_G/A	ARID1A	5.41%	74	non_synonymous_codon
22	DM	Primary_ DNA	17_7574029_G/A	TP53	4.26%	94	non_synonymous_codon
22	DM	Primary_ DNA	16_50785738_C/A	CYLD	5.19%	77	non_synonymous_codon
22	DM	Primary_	17_15983923_C/A	NCOR1	5.06%	79	stop_gained

		DNA					splice_region_variant
23	DM	Primary_ DNA	17_7578406_G/A	TP53	72.15%	299	non_synonymous_codon
23	DM	Primary_ DNA	7_151854917_C/T	MLL3	61.97%	234	stop_gained
23	DM	Primary_ DNA	X_44732930_G/T	KDM6A	44.39%	196	stop_gained
23	DM	Primary_ DNA	9_133760430_C/G	ABL1	19.29%	337	non_synonymous_codon
23	DM	Primary_ DNA	2_202131265_T/A	CASP8	65.77%	222	non_synonymous_codon
24	DM	Primary_ DNA	17_38569734_C/A	TOP2A	3.67%	109	non_synonymous_codon splice_region_variant
24	DM	Primary_ DNA	17_29557285_C/T	NF1	6.06%	66	non_synonymous_codon
26	DM	Primary_ DNA	17_29585418_C/-	NF1	41.21%	381	frameshift_variant
26	DM	Primary_ DNA	14_105246551_G/ A	AKT1	47.93%	180	non_synonymous_codon splice_region_variant
26	DM	Primary_ DNA	13_48954207_A/G	RB1	11.76%	34	non_synonymous_codon
26	DM	Primary_ DNA	5_176719096_A/C	NSD1	10.60%	302	non_synonymous_codon
28	DM	Primary_ DNA	12_49445241_A/G	MLL2	4.60%	87	non_synonymous_codon
28	DM	Primary_ DNA	2_30143189_G/A	ALK	5.71%	70	non_synonymous_codon
28	DM	Primary_ DNA	12_49445206_C/T	MLL2	9.30%	43	non_synonymous_codon
28	DM	Primary_ DNA	6_161519378_C/T	MAP3K4	5.43%	92	non_synonymous_codon
28	DM	Primary_ DNA	17_7574003_C/T	TP53	70.00%	75	stop_gained
28	DM	Primary_ DNA	10_89720809_AAC A/-	PTEN	63.28%	256	frameshift_variant
28	DM	Primary_ DNA	4_1809338_G/A	FGFR3	6.12%	98	non_synonymous_codon
30	CTL	Primary_ DNA	17_41242980_AG/-	BRCA1	7.59%	237	frameshift_variant
30	CTL	Primary_ DNA	17_7578525_C/G	TP53	11.89%	248	non_synonymous_codon
30	CTL	Primary_ DNA	11_125503091_C/T	CHEK1	7.50%	560	non_synonymous_codon
30	CTL	Primary_ DNA	3_178936091_G/A	PIK3CA	6.68%	577	non_synonymous_codon
30	CTL	Primary_ DNA	7_151900103_CT/-	MLL3	7.98%	489	frameshift_variant
30	CTL	Primary_ DNA	13_32971098_C/A	BRCA2	5.58%	556	non_synonymous_codon
32	CTL	Primary_ DNA	9_133760688_CT/-	ABL1	13.16%	190	frameshift_variant
36	DM	Primary_	17_7577543_G/T	TP53	39.45%	758	non_synonymous_codon

		DNA					
36	DM	Primary_DNA	7_128848631_C/A	SMO	26.80%	1112	non_synonymous_codon
36	DM	Primary_DNA	9_135796754_C/T	TSC1	35.15%	549	stop_gained
37	CTL	Primary_DNA	5_67593331_G/C	PIK3R1	7.59%	1185	non_synonymous_codon
37	CTL	Primary_DNA	17_7579591_-/AG	TP53	17.99%	817	splice_region_variant frameshift_variant
38	DM	Primary_DNA	20_57428740_G/A	GNAS	3.03%	132	non_synonymous_codon
38	DM	Primary_DNA	5_56177812_GA/-	MAP3K1	20.56%	180	frameshift_variant
38	DM	Primary_DNA	5_56183241_-/TT	MAP3K1	25.49%	306	frameshift_variant
38	DM	Primary_DNA	2_209113112_G/A	IDH1	15.87%	86	non_synonymous_codon
38	DM	Primary_DNA	1_120484314_C/T	NOTCH2	3.95%	177	non_synonymous_codon
38	DM	Primary_DNA	3_142188955_G/A	ATR	6.15%	179	non_synonymous_codon
38	DM	Primary_DNA	9_98209212_G/A	PTCH1	5.97%	67	non_synonymous_codon
38	DM	Primary_DNA	13_32928997_G/A	BRCA2	5.88%	85	splice_acceptor_variant
38	DM	Primary_DNA	22_41573612_G/A	EP300	4.55%	88	non_synonymous_codon
38	DM	Primary_DNA	17_29557915_G/A	NF1	3.66%	164	non_synonymous_codon
38	DM	Primary_DNA	10_43609021_G/A	RET	4.46%	157	non_synonymous_codon
38	DM	Primary_DNA	2_29519920_C/T	ALK	5.17%	116	stop_gained
38	DM	Primary_DNA	20_57484420_C/T	GNAS	33.05%	239	non_synonymous_codon
38	DM	Primary_DNA	3_178936082_G/A	PIK3CA	16.45%	3026	non_synonymous_codon
38	DM	Primary_DNA	3_142286928_G/A	ATR	6.47%	201	non_synonymous_codon
40	DM	Primary_DNA	10_89692837_T/A	PTEN	23.24%	1700	non_synonymous_codon
41	CTL	Primary_DNA	7_152009018_C/T	MLL3	32.02%	534	stop_gained
43	CTL	Primary_DNA	3_178936082_G/A	PIK3CA	16.45%	3026	non_synonymous_codon
45	CTL	Primary_DNA	20_31019199_G/A	ASXL1	13.08%	1185	non_synonymous_codon
46	LRR	Primary_DNA	3_178936091_G/A	PIK3CA	6.68%	577	non_synonymous_codon
47	CTL	Primary_DNA	13_32936740_G/A	BRCA2	17.43%	895	stop_gained
47	CTL	Primary_	17_7577120_G/A	TP53	30%	150	non_synonymous_codon

		DNA					
47	CTL	Primary_ DNA	3_178936091_G/A	PIK3CA	6.68%	577	non_synonymous_codon
48	LRR	Primary_ DNA	10_8115972_-/A	GATA3	9.69%	413	frameshift_variant
48	LRR	Primary_ DNA	10_8111514_-/G	GATA3	8.25%	824	frameshift_variant
48	LRR	Primary_ DNA	22_41564778_T/C	EP300	4.84%	475	non_synonymous_codon
51	LRR	Primary_ DNA	3_178952085_A/G	PIK3CA	20.96%	225	non_synonymous_codon
51	LRR	Primary_ DNA	17_7577085_G/A	TP53	37.35%	415	non_synonymous_codon
51	LRR	Primary_ DNA	X_66765243_GCA/-	AR	6.23%	289	inframe_codon_loss
51	LRR	Primary_ DNA	3_138664610_C/T	FOXL2	9.52%	42	non_synonymous_codon
51	LRR	Primary_ DNA	17_29676248_C/T	NF1	41.51%	489	stop_gained
53	LRR	Primary_ DNA	7_148511068_C/T	EZH2	18.11%	961	stop_gained
53	LRR	Primary_ DNA	5_149499590_G/A	PDGFRB	29.15%	981	non_synonymous_codon
54	LRR	Primary_ DNA	13_28608541_C/T	FLT3	4.44%	90	non_synonymous_codon
54	LRR	Primary_ DNA	13_32906981_G/A	BRCA2	9.09%	44	non_synonymous_codon
54	LRR	Primary_ DNA	9_98209236_C/T	PTCH1	5.45%	110	non_synonymous_codon
54	LRR	Primary_ DNA	X_76937963_C/G	ATRX	74.07%	81	non_synonymous_codon
54	LRR	Primary_ DNA	2_48010488_G/A	MSH6	44.59%	74	non_synonymous_codon
54	LRR	Primary_ DNA	5_176638401_G/A	NSD1	4.30%	93	non_synonymous_codon
54	LRR	Primary_ DNA	5_149450132_A/G	CSF1R	5.28%	265	non_synonymous_codon splice_region_variant
54	LRR	Primary_ DNA	7_113558612_G/A	PPP1R3 A	8.11%	74	non_synonymous_codon
54	LRR	Primary_ DNA	7_113519964_T/G	PPP1R3 A	4.55%	88	non_synonymous_codon
54	LRR	Primary_ DNA	2_141643727_G/A	LRP1B	8%	50	non_synonymous_codon
54	LRR	Primary_ DNA	2_141116447_C/A	LRP1B	5.03%	159	non_synonymous_codon
54	LRR	Primary_ DNA	22_41548008_A/G	EP300	43.30%	97	non_synonymous_codon
54	LRR	Primary_ DNA	9_139391550_C/T	NOTCH1	4.30%	93	non_synonymous_codon
55	CTL	Primary_ DNA	17_7577098_A/C	TP53	25.63%	437	non_synonymous_codon
55	CTL	Primary_	16_3820836_C/G	CREBBP	27.90%	785	non_synonymous_codon

		DNA					
55	CTL	Primary_DNA	3_178936091_G/A	PIK3CA	6.68%	577	non_synonymous_codon
56	LRR	Primary_DNA	10_8111522_-/T	GATA3	34.35%	1936	frameshift_variant
57	CTL	Primary_DNA	1_65301077_T/C	JAK1	24.26%	940	splice_donor_variant
59	LRR	Primary_DNA	2_29416692_G/A	ALK	3.74%	107	non_synonymous_codon
59	LRR	Primary_DNA	9_139391593_G/A	NOTCH1	7.02%	57	non_synonymous_codon
59	LRR	Primary_DNA	17_29490381_C/T	NF1	5.71%	70	non_synonymous_codon
60	CTL	Primary_DNA	12_49418600_C/G	MLL2	5.27%	550	non_synonymous_codon
60	CTL	Primary_DNA	9_98221968_A/G	PTCH1	38.97%	916	non_synonymous_codon
61	LRR	Primary_DNA	16_23625324_G/T	PALB2	17.69%	616	splice_donor_variant
61	LRR	Primary_DNA	17_7577034_G/C	TP53	7.48%	615	non_synonymous_codon
62	CTL	Primary_DNA	10_89692982_-/G	PTEN	5.94%	539	frameshift_variant
62	CTL	Primary_DNA	13_49033826_-/T	RB1	13.31%	526	splice_region_variant frameshift_variant
62	CTL	Primary_DNA	17_7579414_G/A	TP53	9%	589	stop_gained
63	LRR	Primary_DNA	5_56176959_AAACC/-	MAP3K1	12.42%	1498	frameshift_variant
63	LRR	Primary_DNA	3_178952085_A/T	PIK3CA	19.85%	1169	non_synonymous_codon
64	CTL	Primary_DNA	3_178936082_G/A	PIK3CA	16.45%	3026	non_synonymous_codon
66	LRR	Primary_DNA	17_7578427_A/G	TP53	27.91%	602	non_synonymous_codon
67	CTL	Primary_DNA	17_7577552_CATG/-	TP53	7.50%	600	frameshift_variant
67	CTL	Primary_DNA	19_15272337_-/C	NOTCH3	13.05%	429	frameshift_variant
68	CTL	Primary_DNA	3_178936092_A/G	PIK3CA	44.04%	445	non_synonymous_codon
68	CTL	Primary_DNA	5_56167802_-/T	MAP3K1	25.94%	902	frameshift_variant
68	CTL	Primary_DNA	5_56177908_-/A	MAP3K1	22.67%	344	frameshift_variant
68	CTL	Primary_DNA	17_15961851_TCAC/-	NCOR1	18.85%	488	frameshift_variant
68	CTL	Primary_DNA	3_178936082_G/A	PIK3CA	16.45%	3026	non_synonymous_codon
70	CTL	Primary_DNA	3_178936082_G/C	PIK3CA	16.36%	1100	non_synonymous_codon
70	CTL	Primary_	13_32937361_-/G	BRCA2	22.81%	399	frameshift_variant

		DNA					
70	CTL	Primary_ DNA	7_151878779_C/T	MLL3	27%	426	non_synonymous_codon

Table S2: Genomic alterations by hormone receptor status in patients with recurrent disease, loco-regional (LRR) or distant metastasis (DM), or controls without any type of recurrence

	**Hormone Positive N=42**			**Hormone Negative N=15**		
	LRR N=12	DM N=12	Control N=18	LRR N=5	DM N=3	Control N=7
TP53	4	3	2	3	2	4
PIK3CA	4	2	8	0	0	1
NF1	2	3	0	2	1	0
PI3K/Akt/mTOR pathway	9	10	12	4	1	3
MAPK pathway	5	5	0	3	1	0

Table S3: Differentially expressed genes between the ER+ and ER- primary tumors

	baseMean	log2FoldChange	lfcSE	stat	P value	P adjusted
ADCY6	2219.7503	1.379212393	0.24354157	5.66314974	1.49E-08	4.39E-06
AFF3	2783.72545	2.004288383	0.25311877	7.91837137	2.41E-15	3.55E-12
AGR2	952.168677	2.948135513	0.32816395	8.98372746	2.62E-19	7.25E-16
AGR3	304.52625	2.950731436	0.33152601	8.90045225	5.56E-19	1.12E-15
ANKRD20A11P	589.03247	1.967884525	0.34796086	5.65547669	1.55E-08	4.53E-06
ANKRD30A	20277.1911	4.250290206	0.35572756	11.9481611	6.64E-33	1.47E-28
APBB2	3894.4279	1.54982241	0.23155192	6.69319597	2.18E-11	1.38E-08
APOA1BP	1335.92075	-2.232331951	0.3339647	-6.684335	2.32E-11	1.43E-08
ARHGEF38	884.324188	1.795871049	0.26077738	6.88660603	5.71E-12	4.36E-09
BMPR1B	1941.13134	2.734510638	0.35784089	7.64169416	2.14E-14	2.79E-11
BTG2	9403.79001	1.714790972	0.28268668	6.06604807	1.31E-09	5.01E-07
BTRC	2392.87971	1.560436632	0.24679031	6.32292512	2.57E-10	1.16E-07
CA12	1040.70235	1.889640063	0.30770505	6.14107595	8.20E-10	3.30E-07
CACNA1D	3127.01716	1.409795851	0.24245126	5.81475978	6.07E-09	2.07E-06
CCDC159	597.399098	1.724234985	0.31412398	5.4890269	4.04E-08	9.76E-06
CLEC3A	854.784108	2.859566698	0.44784619	6.38515359	1.71E-10	7.90E-08
CPB1	2231.32712	3.087347907	0.42593496	7.24840214	4.22E-13	3.89E-10
CPEB4	4277.37995	1.24642709	0.20067731	6.21110128	5.26E-10	2.20E-07
CXXC5	2164.6255	1.997910992	0.30564566	6.53669021	6.29E-11	3.57E-08
CYP2B7P	1036.02398	2.100508982	0.30426746	6.90349541	5.07E-12	4.01E-09
CYP4Z2P	1103.8902	2.266920367	0.337171	6.72335508	1.78E-11	1.16E-08
ECM2	1435.04925	1.551000755	0.27329679	5.67515165	1.39E-08	4.25E-06
ELF5	534.502954	-1.988072478	0.32401276	-6.1357846	8.47E-10	3.35E-07
ELP2	2024.98527	1.771007864	0.29966727	5.9099143	3.42E-09	1.26E-06
EN1	1057.62697	-2.416622406	0.35772817	-6.7554714	1.42E-11	9.55E-09
ENPP1	1347.85685	1.708031776	0.27606015	6.18717261	6.13E-10	2.51E-07
ERBB4	2632.24529	1.545014619	0.22759933	6.78830909	1.13E-11	8.11E-09
ERLIN2	1268.92385	1.465828241	0.26417288	5.54874622	2.88E-08	7.83E-06
ESR1	7486.64945	3.254195817	0.30735212	10.5878424	3.39E-26	1.88E-22
EVL	1756.99113	1.48970789	0.26855518	5.54712039	2.90E-08	7.83E-06
F2RL2	1053.28264	1.840171262	0.28624519	6.42865402	1.29E-10	6.20E-08
FAM198B	1066.14525	1.581863389	0.27521016	5.74783799	9.04E-09	2.94E-06
FAM214A	2516.13387	1.71830957	0.23192613	7.40886582	1.27E-13	1.26E-10
FOXA1	2098.29081	2.679955373	0.24921277	10.7536842	5.69E-27	4.20E-23
FOXC1	3182.50113	-2.066935166	0.30287718	-6.8243343	8.83E-12	6.52E-09
FSIP1	1323.14823	2.155202552	0.29091945	7.40824502	1.28E-13	1.26E-10
GABRP	1379.21592	-3.429612852	0.34344407	-9.9859429	1.76E-23	7.78E-20
GATA3	5747.70923	2.384297176	0.26641676	8.9495013	3.57E-19	8.79E-16
GREB1	3010.99667	1.390803739	0.24819055	5.60377396	2.10E-08	5.95E-06
GTF2H2B	158.79957	2.423662003	0.38421098	6.30815386	2.82E-10	1.25E-07
HOXC10	1934.71638	1.525251018	0.27567635	5.53275981	3.15E-08	8.12E-06
HSPB6	1062.10869	1.621139306	0.29536775	5.48854544	4.05E-08	9.76E-06
IL6ST	7277.2637	1.827194672	0.28035923	6.51733373	7.16E-11	3.87E-08
INPP4B	1500.66622	1.55435495	0.26821707	5.79513814	6.83E-09	2.29E-06
KAT6B	6969.68551	1.128556819	0.20382946	5.53677001	3.08E-08	8.03E-06

KHDRBS3	406.306772	-1.458432279	0.25297701	-5.7650784	8.16E-09	2.70E-06
LDLRAD4-AS1	3050.78779	2.696252271	0.35789431	7.53365506	4.93E-14	6.07E-11
LINC00993	17.5382241	3.374092207	0.49814656	6.7732922	1.26E-11	8.71E-09
LOC646214	1150.36478	1.498965753	0.27133881	5.5243323	3.31E-08	8.32E-06
LYPD6B	221.028771	2.014733504	0.35511601	5.67345155	1.40E-08	4.25E-06
MCCC2	1300.87625	1.908777399	0.29557823	6.45777404	1.06E-10	5.47E-08
MLPH	1998.9765	1.922421045	0.29444631	6.52893577	6.62E-11	3.67E-08
MMP7	438.55389	-3.104992855	0.34460236	-9.0103644	2.05E-19	6.50E-16
MRAS	676.06477	-1.411520613	0.24597874	-5.7383847	9.56E-09	3.07E-06
MUC1	7741.74738	1.873191503	0.29292195	6.39484851	1.61E-10	7.57E-08
MUC6	29345.5004	1.905101572	0.31135457	6.11875263	9.43E-10	3.66E-07
NBEA	2958.85047	1.063101976	0.19171256	5.54529113	2.93E-08	7.83E-06
NFKBIZ	4114.45026	2.117911382	0.29608264	7.15310898	8.48E-13	7.51E-10
PIP	1004.95583	2.620784753	0.40349918	6.49514275	8.30E-11	4.37E-08
POTEKP	3312.61493	4.549255916	0.39582871	11.4929913	1.43E-30	1.58E-26
PREX1	2598.19439	1.298261346	0.22779926	5.6991465	1.20E-08	3.76E-06
PROM1	917.228367	-2.576158438	0.29015791	-8.8784706	6.78E-19	1.25E-15
RAB11FIP1	3492.06809	1.752441478	0.29651612	5.91010535	3.42E-09	1.26E-06
RBM47	2148.86361	1.387735296	0.24898485	5.57357313	2.50E-08	6.91E-06
RERG	534.457904	1.441701388	0.25454968	5.66373287	1.48E-08	4.39E-06
RHOB	2603.0228	1.583570382	0.2698324	5.86871851	4.39E-09	1.57E-06
ROPN1	120.538041	-2.14768751	0.30424518	-7.0590683	1.68E-12	1.37E-09
SCGB2A2	2756.23479	2.827172178	0.44997578	6.28294306	3.32E-10	1.41E-07
SCNN1A	1154.0103	1.453978588	0.2630902	5.52654038	3.27E-08	8.31E-06
SCUBE2	3422.0394	2.372642938	0.31802257	7.46061186	8.61E-14	9.87E-11
SFRP1	419.429686	-1.919937144	0.29825954	-6.4371358	1.22E-10	5.99E-08
SLC39A6	15423.9859	2.702613694	0.31610674	8.54968706	1.23E-17	2.10E-14
SLC4A8	1530.24689	1.481198158	0.22956012	6.45233236	1.10E-10	5.54E-08
SLC6A14	511.967683	-1.551492883	0.28152484	-5.5110337	3.57E-08	8.88E-06
SLC7A8	2582.71666	1.607302306	0.27386582	5.86894086	4.39E-09	1.57E-06
SLC9A3R1	1814.67714	1.724664589	0.29434066	5.85941679	4.64E-09	1.63E-06
SPRYD3	744.575076	1.355617594	0.24242114	5.59199413	2.24E-08	6.29E-06
STC2	2148.87229	2.182284303	0.32943399	6.62434463	3.49E-11	2.09E-08
SYT1	798.022458	1.550287304	0.28157027	5.50586298	3.67E-08	9.04E-06
SYTL2	4340.92386	1.803374862	0.27343529	6.59525288	4.25E-11	2.47E-08
TBC1D9	4415.67398	2.200517618	0.24724828	8.90003188	5.58E-19	1.12E-15
TBX3	1231.94275	1.326199515	0.23151364	5.72838618	1.01E-08	3.21E-06
TENC1	1560.95689	1.318014117	0.22537959	5.84797452	4.98E-09	1.72E-06
TFF1	1527.1452	3.174247079	0.38965322	8.14633871	3.75E-16	5.93E-13
TFF3	945.718972	3.306843987	0.352495	9.38125085	6.52E-21	2.41E-17
TM4SF18	702.807149	2.327417716	0.37007783	6.28899525	3.20E-10	1.39E-07
TMEM198B	4465.75241	2.552294634	0.35727747	7.14373234	9.08E-13	7.74E-10
TOB1	3143.04805	1.416489795	0.25083045	5.64720023	1.63E-08	4.69E-06
WIBG	505.507862	1.97964582	0.35738525	5.53924874	3.04E-08	8.01E-06

XBP1	6837.56488	2.370984598	0.30289418	7.82776534	4.97E-15	6.87E-12
ZNF552	4235.24724	1.758620651	0.23586211	7.45613896	8.91E-14	9.87E-11
ZNF703	2686.41651	2.390662369	0.32285073	7.40485364	1.31E-13	1.26E-10

Table S4: Tumor subtype and IHC data for Ki-67, CC3 and PTEN results

Patient ID	Case or Control	Hormone receptor positive (either ER, PR or both+)	Subtype	ki67 primary exact (%)	CC3 results	PTEN results
1	DM	Yes	Luminal B	40	Positive	Positive
2	Control	Yes	Luminal A	10	Negative	Negative
3	DM	No	Triple negative	10	Negative	Negative
4	Control	No	Triple negative	35	Negative	Negative
5	Control	No	Triple negative	1	Positive	NA
6	DM	Yes	Luminal A	10	Negative	Positive
7	Control	Yes	Luminal A	10	Negative	Positive
8	DM	No	Triple negative	20	Negative	Positive
9	Control	No	Triple negative	25	Negative	Negative
10	LRR	No	Triple negative	75	Negative	Negative
11	Control	No	Triple negative	60	Positive	Positive
12	LRR	Yes	Luminal A	10	Negative	Positive
13	Control	Yes	Luminal A	1	Negative	Positive
14	LRR	Yes	Luminal B	35	Negative	Positive
15	Control	Yes	Luminal A	1	Negative	Positive
16	LRR	Yes	Luminal B	66	Negative	Positive
17	Control	Yes	Luminal B	5	Negative	Positive
18	LRR	Yes	Luminal A	1	Negative	Negative
19	Control	Yes	Luminal A	0	Negative	Positive
20	LRR	Yes	Luminal A	2	Negative	Positive
21	Control	Yes	Luminal A	5	Negative	Negative
22	DM	Yes	Luminal B	90	Negative	Positive
23	DM	No	Triple negative	80	Negative	Negative
24	DM	Yes	Luminal B	30	Negative	Negative
25	Control	No	Triple negative	85	Negative	Positive

26	DM	Yes	Luminal A	5	Negative	Positive
27	DM	Yes	Luminal A	1	Negative	Positive
28	DM	Yes	Luminal A	1	Negative	Negative
29	DM	No	Triple negative	80	Negative	Positive
30	Control	No	Triple negative	30	Negative	Negative
31	DM	Yes	Luminal B	25	Negative	Negative
32	Control	Yes	Luminal A	5	Negative	Positive
33	DM	Yes	Luminal B	20	Negative	Negative
34	DM	Yes	Luminal A	15	NA	Positive
35	Control	Yes	Luminal A	10	Negative	Positive
36	DM	Yes	Luminal B	20	Negative	Positive
37	Control	Yes	Luminal B	15	Negative	Positive
38	DM	Yes	Luminal A	1	Negative	Positive
39	Control	Yes	Luminal A	15	Negative	Positive
40	DM	Yes	Luminal B	15	Negative	Positive
41	Control	Yes	Luminal A	10	Negative	Positive
42	DM	Yes	Luminal A	0	Negative	Negative
43	Control	Yes	Luminal A	1	Negative	Positive
44	DM	Yes	Luminal A	3	Negative	Negative
45	Control	Yes	Luminal B	30	Negative	Positive
46	LRR	Yes	Luminal A	1	Negative	Negative
47	Control	Yes	Luminal B	20	Negative	Negative
48	LRR	Yes	Luminal A	1	Negative	Negative
49	Control	Yes	Luminal A	10	Negative	Negative
50	Control	Yes	Luminal A	0	Negative	Negative
51	LRR	Yes	Luminal A	15	Negative	Negative
52	Control	Yes	Luminal B	5	Negative	Negative
53	LRR	Yes	Luminal A	2	Negative	Negative
54	LRR	No	Triple negative	65	Negative	Negative
55	Control	Yes	Luminal A	1	Negative	Negative
56	LRR	Yes	Luminal A	10	Negative	Negative
57	Control	Yes	Luminal B	35	Negative	Negative
58	LRR	Yes	Luminal A	5	Negative	Positive
59	LRR	No	Triple negative	70	Positive	Negative
60	Control	No	Triple negative	50	Negative	Negative
61	LRR	No	Triple negative	45	Negative	Negative
62	Control	No	Triple negative	1	Negative	Negative
63	LRR	Yes	Luminal B	30	Negative	Positive
64	Control	Yes	Luminal A	1	Negative	Positive
65	Control	Yes	Luminal A	0	Negative	Negative
66	LRR	No	Triple negative	40	Negative	Negative
67	Control	No	Triple negative	35	Negative	Negative
68	Control	Yes	Luminal A	1	Negative	Positive
69	DM	Yes	Luminal A	15	Negative	Negative
70	Control	Yes	Luminal A	1	Negative	Negative
71	Control	Yes	Luminal B	0	Negative	Negative
72	DM	Yes	Luminal A	5	Negative	Positive
73	Control	Yes	Luminal A	0	Negative	Negative

74	DM	Yes	Luminal A	0	Negative	Positive
75	Control	Yes	Luminal A	5	Negative	Positive
76	Control	Yes	Luminal A	5	Negative	Positive
77	Control	Yes	Luminal B	0	Negative	Negative
78	Control	Yes	Luminal A	0	Negative	Positive
79	Control	No	Triple negative	1	Negative	Negative
80	LRR	Yes	Luminal B	30	Negative	Positive
81	Control	Yes	Luminal A	0	Negative	Positive
82	DM	Yes	Luminal B	20	Negative	Positive
83	DM	Yes	Luminal B	10	Negative	Positive
84	DM	Yes	Luminal A	1	Negative	Positive
85	DM	Yes	Luminal A	10	Negative	Positive
86	LRR	Yes	Luminal A	0	Negative	Positive
87	DM	Yes	Luminal B	2	Negative	Positive
88	LRR	Yes	Luminal B	15	Negative	Positive
89	LRR	Yes	Luminal B	20	Negative	Positive
90	LRR	No	Triple negative	15	Negative	Positive
91	DM	Yes	Luminal A	5	Negative	Positive
92	LRR	Yes	Luminal B	35	Negative	Positive
93	LRR	No	Triple negative	80	Negative	Negative
94	LRR	Yes	Luminal B	25	Negative	Positive
95	Control	Yes	Luminal A	15	Negative	Negative
96	Control	Yes	Luminal B	20	Negative	Positive
97	DM	No	Triple negative	80	Positive	Negative
98	Control	Yes	Luminal A	5	Positive	Positive
99	Control	Yes	Luminal A	1	Negative	Positive
100	Control	Yes	Luminal A	5	Negative	Positive
101	Control	Yes	Luminal B	20	Negative	Positive
102	Control	Yes	Luminal A	5	Negative	Positive
103	LRR	Yes	Luminal B	65	Negative	Positive
104	DM	Yes	Luminal B	55	Negative	Positive
105	Control	Yes	Luminal B	90	Negative	Positive
106	LRR	Yes	Luminal A	10	Negative	Positive
107	LRR	Yes	Luminal B	60	Negative	Positive
108	LRR	No	Triple negative	45	Negative	Positive
109	DM	No	Triple negative	10	Negative	Negative
110	DM	No	Triple negative	85	Negative	Positive
111	DM	Yes	Luminal B	35	Negative	Positive
112	DM	Yes	Luminal B	85	Negative	Negative
113	LRR	Yes	Luminal B	5	Negative	Positive
114	LRR	Yes	Luminal B	30	Negative	Positive
115	LRR	No	Triple negative	25	Negative	Negative
